# Comparison of Police Data on Animal Cruelty and the Perception of Animal Welfare NGOs in Hungary

**DOI:** 10.3390/ani13071224

**Published:** 2023-03-31

**Authors:** Gábor Lorászkó, Szilvia Vetter, Bence Rácz, Péter Sótonyi, László Ózsvári

**Affiliations:** 1 Department of Anatomy and Histology, University of Veterinary Medicine Budapest, H-1078 Budapest, Hungary; loraszko.gabor@univet.hu (G.L.); racz.bence@univet.hu (B.R.); sotonyi.peter@univet.hu (P.S.); 2 Center for Animal Welfare, University of Veterinary Medicine Budapest, H-1078 Budapest, Hungary; vetter.szilvia@univet.hu; 3 Department of Veterinary Forensics and Economics, University of Veterinary Medicine Budapest, H-1078 Budapest, Hungary

**Keywords:** animal welfare, animal protection, animal cruelty, police, NGOs, survey, Hungary

## Abstract

**Simple Summary:**

The public often expresses a high level of dissatisfaction with the work of the animal welfare authorities. On the one hand, we investigated the police’s procedural practices in relation to animal cruelty and on the other hand, we asked animal protection non-governmental organisations (NGOs) for their views on animal cruelty and the related police and judicial activities. We found that the police generally act quickly and with caution. However, the most sensitive section of the public is practically unaware of animal cruelty and forms its opinion on the basis of its concerns. Although most police officers consider the crime of animal cruelty to be somewhat more important than the average, the vast majority of animal welfare NGOs consider this crime to be extremely important. Three emotions play a prominent role for animal welfare NGOs in relation to animal cruelty: anger, disgust and sadness. For this very reason, it would be beneficial if the police were to provide more detailed information to the public, because the prejudices that give rise to dissatisfaction with them are largely unjustified.

**Abstract:**

Animal cruelty has been a criminal offence in Hungary since 2004 and the legislator has tightened and differentiated the regulations in several waves since then. However, it is not an exaggeration to say that the public is often impatient and dissatisfied with the actions of the authorities in relation to animal cruelty. In our research, based on the data of the Criminal Investigation Department of the National Police Headquarters, we examined the opinions of 99 out of a total of 155 police stations in Hungary whose staff currently working there had experience in dealing with animal cruelty. The investigators gave their opinion on a total of 1169 cases in which some kind of police action was taken, either following a report to the police or as a result of their own investigative actions. In another survey, we questioned those members of society who are most committed to animal protection using a self-completion questionnaire. The questionnaire sent to the 116 Hungarian animal welfare non-governmental organisations (NGOs) on the publicly available lists was also posted for a short period on the social networking site of NGO activists. Among those who responded, a total of 150 identified as active participants in the animal protection work of these NGOs. The picture of the police treatment of animal cruelty, as perceived by NGOs working in the field of animal protection, is significantly less favourable than suggested by the police data. According to the official data, 77.7% of reports initiated an investigation, while the vast majority of animal welfare activists (81.3%) suspects that only 25% of the reports result in action by the prosecuting authority.

## 1. Introduction

While animal abuse is widespread worldwide, its true extent is largely unknown [1]. Opinions on the causes are also divided and sometimes contradictory [2], and much of the research has been carried out using non-representative samples [3]. Some sources indicate a steady increase in the number of cases in Australia, probably due to a decrease in latency in the case of dogs and an increased awareness of animal welfare among the population [4].

The protection of animals has fundamentally changed over the last fifty years, but since they cannot protect their own interests, others do it for them. The roots of this development lasting to this day [5] date back to the XVIIIth century [6], although initially, crimes against animals and nature were considered as a breach of obligations towards people [7]. At the same time, Kant recognized that, on the one hand, someone who is cruel to animals may treat humans in the same way, and on the other hand, getting to know animals better may lead to loving them. Moreover, for almost fifty years, more and more people believe that, in order to protect the ecological balance of nature, even inanimate objects need to be granted the right to legal self-defence, citing trees and companies as analogous examples [8]. Animal rights activists are characterised by idealism [9]. The concepts of animal cruelty and animal welfare are not uniform in the literature and require a complex approach [10,11,12,13,14,15,16].

Based on the responses from 78 of the member countries of the World Organization for Animal Health (OIE), the most important issue for animal protection was considered to be the occurrence of stray dogs [17]. Animal protection work is carried out in different divisions in each country by civil society organisations, police, prosecutors, official veterinarians and other state bodies and organisations.

One reason for public and professional concern about the investigation and prosecution of animal cruelty is the well-documented connection between animal cruelty and interpersonal violence [10,16,18,19,20,21].

In Hungary, the concept of animal cruelty is applied by two acts: the so-called Animal Protection Act (since 1999) and the Criminal Code (since 2004). In order to establish the technical criteria for the criminal offence of animal cruelty under the Criminal Code, the following questions must be answered:

1. Whether it was committed by ill-treatment or by mistreatment;
2. Whether that action was justified;
3. Whether it involved a risk of permanent damage to the animal’s health or its death;
4. Whether particular suffering occurred.

The criminal offence of animal cruelty can be established where an act of cruelty or the treatment of an animal is both unjustified and involves a risk of permanent harm to the animal’s health or its death. As an aggravating circumstance, the punishment scale is increased if particular suffering of the animal occurred. The so-called Animal Protection Act provides a broader definition of animal cruelty (e.g., not limited to the possibility of permanent damage to health, but covering all damage to health, and the possibility of establishing the existence of lasting fear even in the absence of damage to health), but its occurrence is not investigated and sanctioned by the police, but by the Animal Protection Office, which is composed of official veterinarians.

When the police receive a complaint or a report, or when the police authority itself detects a crime, it first examines whether a criminal offence can be suspected. If so, a procedure is initiated (investigation, interviewing witnesses, etc.) and, if the suspicion is confirmed, the case is referred to the prosecuting authority and, if the prosecutor concludes there are sufficient grounds, the suspect is indicted and brought before a court. If the suspicion is not substantiated, the case is closed without any further action and the proceedings are terminated.

One of the goals of our study was to survey the police practice in criminal investigations initiated on suspicion of animal cruelty. As another goal, we researched the opinions of animal welfare activists from animal welfare non-governmental organisations (NGOs) on animal cruelty and the actions of the authorities against animal cruelty.

## 2. Materials and Methods

In response to our request, the Criminal Investigation Department of the National Police Headquarters sent 23 questions to all 155 police stations in Hungary and we received the answers of 99 of them, whose current staff members have taken some kind of police action (receiving a complaint or report, taking a witness testimony, taking an action, ordering, conducting or terminating an investigation, forwarding the file to the prosecution; hereinafter referred to as “case” or “investigation”) in connection with the criminal offence of animal cruelty. The responses concern the investigative experience of 1169 cases in the period between 01 July 2013 and 31 December 2021. One questionnaire per police station was returned to us. The questionnaire can be found in the Supplementary Materials.

**Figure 1:**
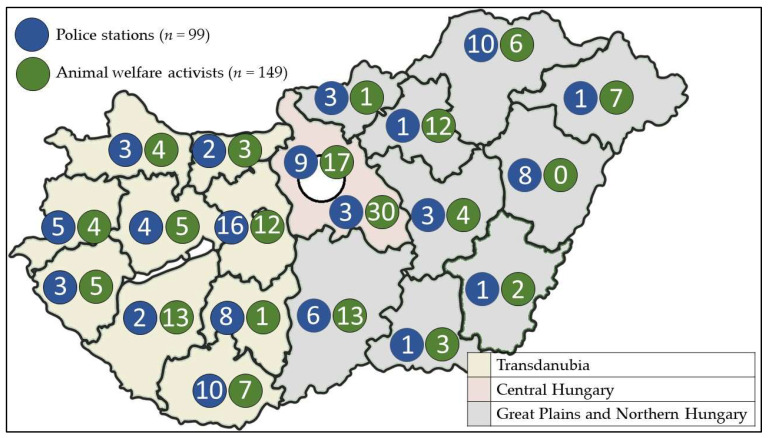
The territorial distribution of the number police stations and that of animal welfare activists filling out the questionnaire.

The number of cases given by each police station ranged from 1 to 100, but responses were taken into account with equal weight and examined by geographical regions (Great Plains and Northern Hungary, Central Hungary and Transdanubia) (Figure 1). The response rate of 63.9% is remarkably high for a self-completion questionnaire; although it varied by region, the absence of significant skews in the data also leads us to believe that the sample is broadly representative and the findings can thus be a sound basis for pragmatic recommendations.

A questionnaire containing 19 questions was sent to all 116 Hungarian animal rights NGOs that had provided their public contact details, and was published on the social networking sites of the ones that cooperated. Between 29 October and 29 December 2021, we received 194 completed questionnaires from organisations. The questionnaire can be found in the Supplementary Materials.

In our analysis, we classified as “active” those who actually undertake animal protection work and those which help civil animal protection with donations (150), based on their relationship with animal protection NGOs. In the evaluation, we only took the responses of active animal welfare activists into account.

It should however be borne in mind that the questionnaire categorized the degree of animal suffering based on the language generally used by the police, not that in the Criminal Code, which is logical considering that in Hungary (as with all Civil Law countries), the decision to indict and, if so, on what specific charge, is the responsibility of the prosecutor based on the police’s criminal investigation, and their actions and perceptions were not researched in the present study.

In the police questionnaire, we asked about the following: area of jurisdiction (county), number of cases experienced, the event that triggered police action, its frequency, the relative (and subjective) importance of the criminal offence of animal cruelty in the eyes of the investigator, the reason for and rate of action taken to refuse to order an investigation, the method of involving a forensic expert, the time taken to initiate and close an investigation, and the time taken before a case is referred to the courts, as well as the proportion of these periods, the difficulties in discovering the facts of the case, the criminal offences associated and the circumstances and conditions likely to increase the efficiency of police work.

To express the frequencies, we have asked respondents to use the adjectives never, exceptionally, rarely, often and most often, and elsewhere never, exceptionally, usually not, usually and always. With regard to the difficulty of examining the factual elements, the adjectives self-evident, easy, somewhat cumbersome and difficult were used for the sake of clarity. For the (subjective) classification of the importance of the criminal offence of animal cruelty, we offered one-sentence formulations for choice; thus, the definition of the category “average” is as follows: “It doesn’t represent an important crime; it’s one of the many to be prosecuted”. The phrases given for the other categories: “I classify it as less important”, “I consider it as one of the more socially important ones”, “I consider it an extremely important crime”. This is intended as a way of assessing the investigators’ emotional attitudes towards animal cruelty. It should also be borne in mind that each police officer in Hungary must investigate any and all types of reported crime so we hypothesise that they are generally more able to stay dispassionate and process-focused than animal activists.

When a person is made a suspect, the outcome of previous prosecutions of that person also become part of the file, which the investigator can see immediately. Investigators were also asked for their observations on suspects’ reactions to being formally accused of the criminal offence of animal cruelty, examining whether there is a difference in the way it is received by people with a criminal record and those without.

In addition to some demographic data from animal welfare NGOs, we asked for their views on the rate at which the police investigate reports or information about animal cruelty, the rate at which the courts convict defendants, the deterrent effect of sentences, how this could be enhanced, the role of biological knowledge in the investigation of animal cruelty cases, what the role of the media and social media is, what their relationship with animal welfare NGOs is and what their opinion is of them, how important a criminal offence they consider animal cruelty, what emotions an animal’s suffering triggers in them, what forms of animal cruelty they consider to be common and difficult to discover, what the situation of animal welfare in Hungary is and how it could be improved.

For the multiple-choice questions, clarifying sentences were generally provided. When examining the emotional reactions of animal welfare activists, only the highest degree of emotion was taken into account for the depiction. In addition to presenting the two groups separately, some aspects of them were also comparable.

To compare the research results statistically, we used a two-sample Z-test, thus examining the differences in distribution. We analysed the survey data according to the regions of the country, and in the case of animal welfare activists, also according to the biological sex of the respondents. The difference was considered significant at *p* < 0.05.

## 3. Results

### 3.1. Initiation of Criminal Investigations

Criminal investigations were initiated by animal welfare NGOs (29.8%), anonymous (26.0%) and self-identified informers (18.9%), various authorities (12.8%) and the police itself (12.6%). Anonymous reporting is significantly rarer in Central Hungary (*p* < 0.05). The police initiated proceedings for 77.7% of the reports; in contrast, the vast majority (81.3%) of the 150 active animal welfare activists who filled out the questionnaire assumed that the investigating authority initiated proceedings in only 25% of the reports. A significantly higher proportion of middle-aged people (*p* = 0.02) and those living in Central Hungary (*p* = 0.01) thought so. According to 15.3% of them, investigations may be initiated in 50% of reports, and 2% of them were of the opinion that it was so in 75% of the cases, and 1.3% assumed that this was always the case. A significantly higher proportion (45.5%) of people under 30 (*p* = 0.01) and those living in Transdanubia (24.1%) assumed that investigations could be initiated based on 50% of the reports (*p* = 0.01).

The vast majority of decisions to initiate criminal investigations (70.8%) were made by the police within a week, and 7.3% of cases were decided after more than a month. There is no significant difference between the regions of the country. An expert was not (41.8%), only very rarely (11.2%) or usually not (26.5%) assigned to decide on the initiation of the procedure. A minority of police stations usually (16.3%) or always (4.1%) used a forensic expert in their decisions.

If the prosecutor decides there is a criminal case to answer, the indictment follows in 1 to 3 months (6.2%), 3 to 6 months (34.0%) or 6 to 12 months (46.4%), but it can take 1 to 1.5 years (9.3%) or even longer (4.1%).

### 3.2. The Importance of the Criminal Offence of Animal Cruelty

**Figure 2:**
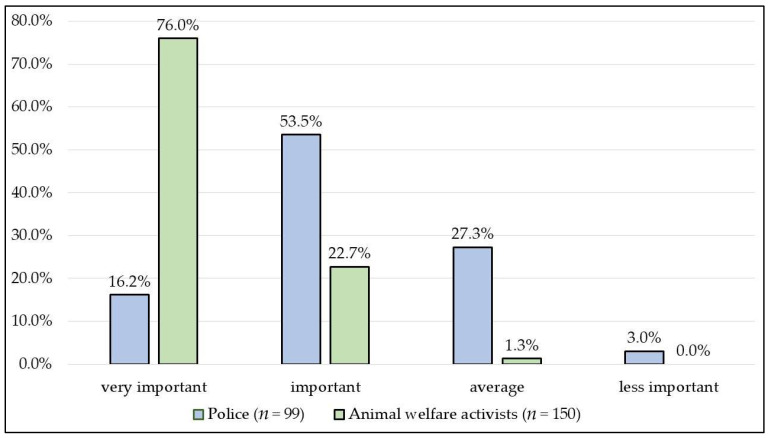
The importance of animal cruelty as a felony, according to police offices and animal welfare activists.

More than two-thirds of police stations (69.7%) considered animal cruelty to be more important than the average crime. The opinion of animal welfare activists on the importance of the felony of animal cruelty proved to be extremely important (76%) or important (22.7%), but significantly more (27.3%) of the investigators considered it an average felony (*p* < 0.0001), and 3% of them considered it less important than the animal welfare activists did. Only 1.3% of animal welfare activists consider animal cruelty to be an average crime (Figure 2).

**Figure 3:**
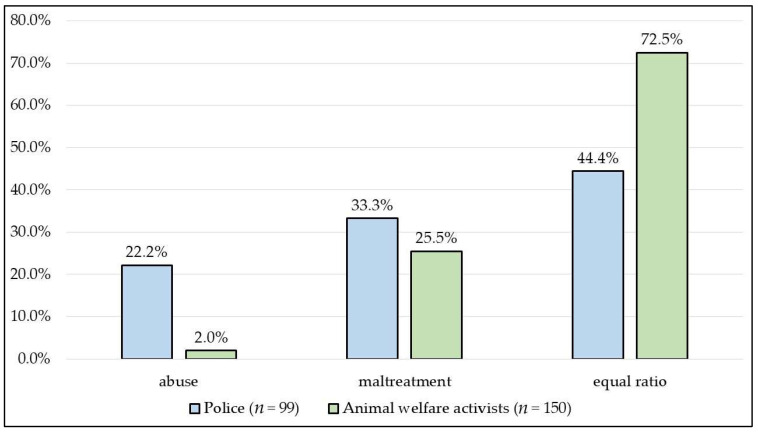
The probability of modus operandi according to police offices and animal welfare activists.

In terms of the probability of the two types of perpetrations occurring, significantly (*p* < 0.0001) fewer animal welfare activists (2.0%) considered abuse to be more frequent than the police (22.2%), and the equal probability of occurrence was estimated to be significantly (*p* < 0.0001) higher by animal welfare activists (72.5%) than the police (44.4%) (Figure 3).

### 3.3. Investigating and Taking Evidence

**Figure 4:**
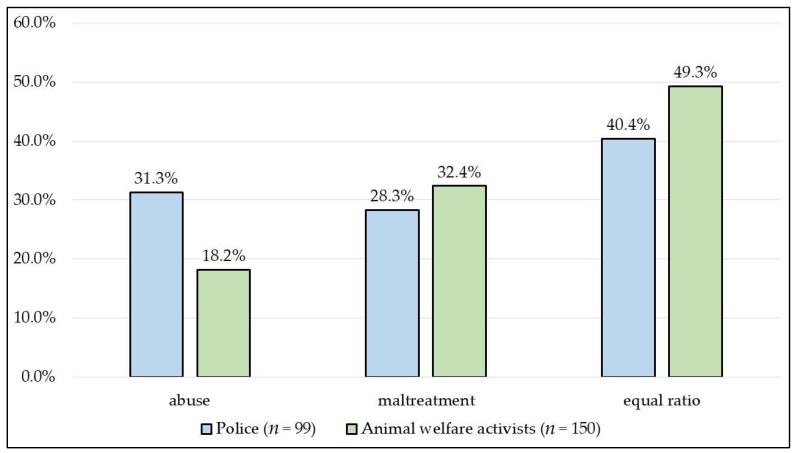
Which form of animal cruelty is more difficult to investigate?

The police (31.3%) considered the investigation of abuse to be significantly more difficult than animal welfare activists (18.2%) believed (*p* = 0.0169) (Figure 4). Among the animal welfare activists, males significantly considered the investigation related to abuse to be more difficult than females (*p* = 0.0159).

**Figure 5:**
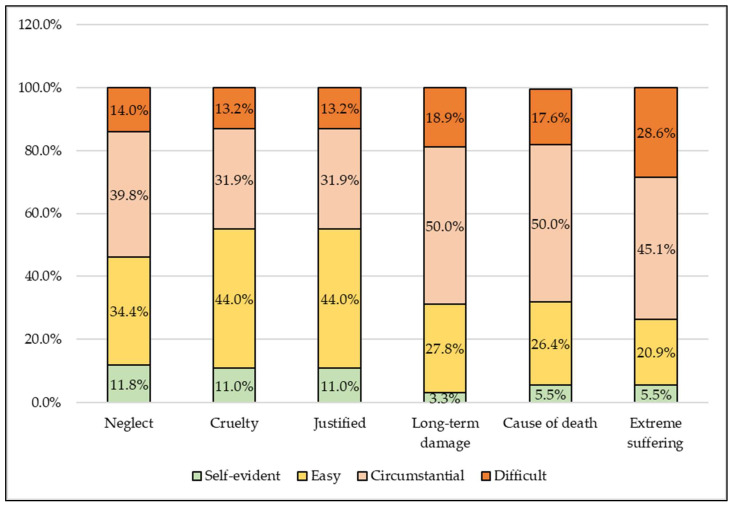
According to the police authorities, how difficult is it to investigate certain elements of the criminal law on animal cruelty? (*n* = 99).

The largest share of police authorities (28.6%) considered the investigation of the particularly burdensome nature of animal suffering difficult, which did differ significantly (*p* < 0.05) from the other parameters (Figure 5).

**Figure 6:**
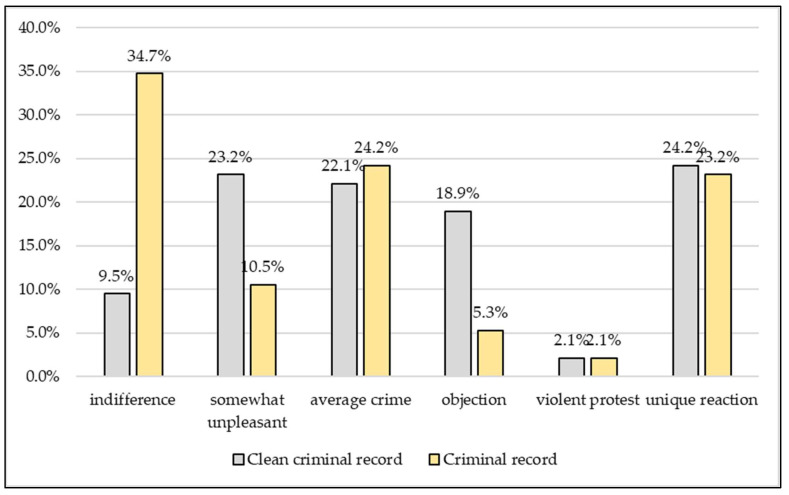
What effect does the suspicion of animal cruelty have on the suspects, depending on whether they have a criminal record or not? (*n* = 99).

Being suspected of the criminal offence of animal cruelty, of becoming a suspect in an investigation, triggered different reactions from the suspects depending on whether they had been previously convicted of a criminal offence. Being suspected of committing the crime of animal cruelty was received with indifference by a significantly (*p* < 0.0001) higher proportion of those with a criminal record (34.7%) than by those with a clean criminal record (9.5%) and a significantly higher proportion protested against the suspicion (*p* = 0.0033) In about a quarter of the cases in both groups, atypical reactions were observed by the police, with a very few but violent (2.1%) protests common to both (Figure 6).

**Figure 7:**
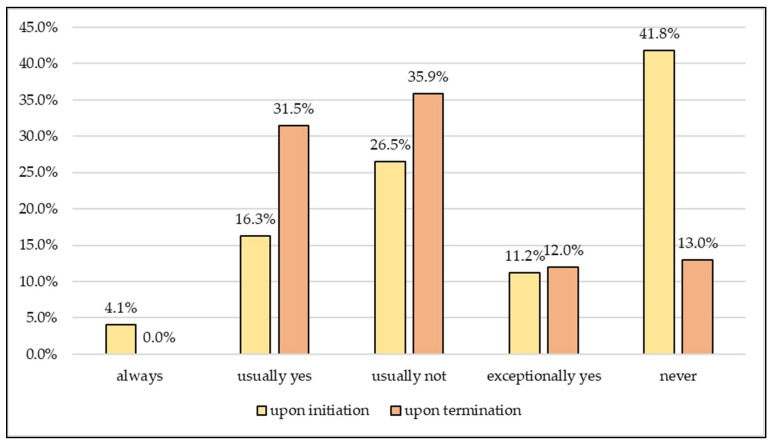
Involvement of forensic experts in the procedure (*n* = 99).

Termination means closure of the criminal investigation with no further police action and without anyone being indicted. An expert was involved significantly (*p* = 0.0104) more often in general upon the termination (31.5%) than the initiation of the procedure (16.3%), and significantly (*p* < 0.0001) fewer police officers bypassed it completely upon termination (13%) than upon initiation (41.8%) (Figure 7). The procedure was terminated significantly (*p* = 0.0234) more often for suspicion of perpetration by mistreatment (32.2%) than for abuse (26.7%). In 31.1% of the police stations, there was no difference between the two forms.

Other crimes are also associated with the crime of animal cruelty (Table 1). The ‘other crime’ category includes fraud, trafficking in human beings, assault, forced labour, document forgery, fish poaching, animal poaching and vandalism.

In the context of conditions that could increase the efficiency of investigators’ work, the police considered cooperation with other authorities (57.6%) and increasing their investigative capacity (57.6%) to be the most important. These two conditions are significantly (*p* < 0.05) more important than possible improvements regarding specialised training (42.4%), more accurate reports (38.4%), forensic experts (31.3%) and technical tools (22.2%). Specialised training was also considered significantly (*p* = 0.0028) more valuable than the improvement of technical equipment.

### 3.4. The Opinion of Animal Welfare NGOs

**Figure 8:**
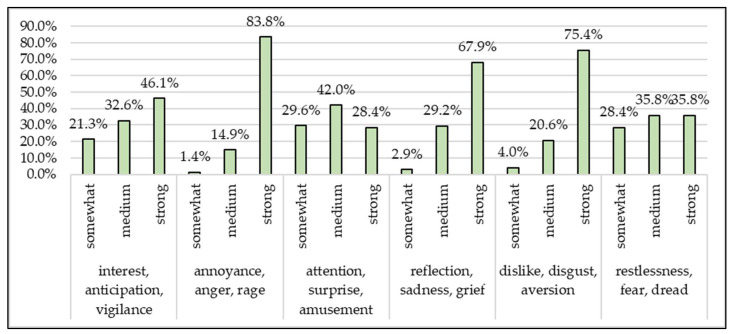
What emotion is activated in animal welfare activists when someone causes suffering to an animal and to what degree? (*n* = 150).

**Figure 9:**
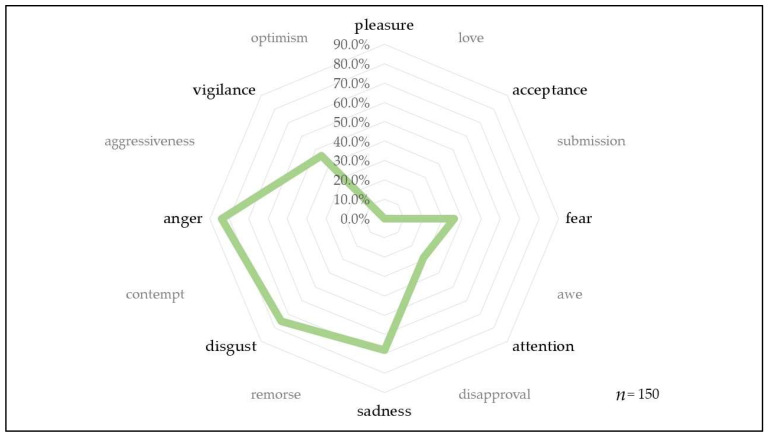
Extent of very strong emotional reactions of animal welfare activists (*n* = 150).

We also examined the emotional reactions of animal welfare activists to animal cruelty (Figure 8 and Figure 9).

When asked about the emotional reactions considered to be very strong, anger and dislike significantly (*p* = 0.0012) exceeded even sadness. Strong anger has a significantly high rate (*p* < 0.0001) amongst women. A high degree of sadness is significantly characteristic (*p* = 0.0158) of females and the older age group, while fear is significantly (*p* = 0.0030) less characteristic of males.

According to the vast majority (90.0%) of animal welfare activists, 25.0% of those brought to court on charges of animal cruelty are convicted. A significantly (*p* < 0.0001) higher proportion of women assessed this conviction rate. According to 6.0% of the respondents, half of the defendants are found guilty, and 2.0% thought that the court hands down a guilty verdict in three quarters of the proceedings. In addition, 2.0% of active animal welfare activists believed that every defendant was convicted. According to the majority of animal welfare activists, punishments have little (18.0%) or no deterrent effect (76.0%) (*p* < 0.0001); 6.0% of them believe that they do. The majority of animal welfare activists (76.5%) would hope that serving prison sentences has a deterrent effect on crime. Significantly more women (*p* = 0.0253) see it this way; men would also find another method appropriate (*p* < 0.0001). Few people (5.4%) believe in the effectiveness of humbling.

The majority of men and women working in animal protection considered the status of protecting animals in Hungary to be low (74%), not significantly different from each other (*p* = 0.4975). This opinion was uniform among the age groups (*p* > 0.05). No significant (*p* > 0.05) differences could be observed between the parts of the country either. In addition, 24% of the respondents considered it of medium standard and 2% as high quality. One third considered the work of NGOs to be extremely valuable, 42% mostly useful, and 24.7% considered only partly valuable. Men are significantly more critical of the work of some animal rights NGOs (*p* = 0.0312). Significantly more people below 30 years of age considered animal protection NGOs to be extremely valuable compared to older people (*p* = 0.0413).

More than a third of animal welfare activists do not (17.0%) or only sometimes (19.0%) considered it important to learn about the physiological needs of animals in procedures related to animal cruelty; 64% think it is necessary. A significantly higher proportion of young people consider professional knowledge to be important than those older than 51 (*p* = 0.0004).

More than a third of animal welfare activists (35.0%) consider the news related to animal cruelty to be an authentic source, suitable for forming an opinion; a slightly higher proportion (42.0%) evaluate before accepting it, and 15% consider it distorted information suitable for influencing people. A significant proportion of men (*p* = 0.0024) consider it a less credible source and significantly more young people require more consideration of the news than older people (*p* < 0.0434). In relation to animal cruelty-related news appearing on social media, only about a tenth (11%) of animal welfare activists did not form a firm opinion; 88% agree that it triggers emotions, and the majority (61%) also comment on it. Men are significantly less likely to form a firm opinion about the news (*p* = 0.0315), and more often they are not even interested (*p* = 0.0019). Compared to young people, both older age groups comment a lot more often when they do not like anything (*p* < 0.0157) or when they hear about an event that causes outrage (*p* < 0.0027). There is no significant difference in the reactions amongst different parts of the country.

## 4. Discussion

Emotions are of crucial importance in the various civil movements [22,23,24,25], which can be measured even by means of mathematics [26], and their role is well known in animal rights activism [27,28,29]. In our research, animal welfare activists felt the strongest the emotions of anger (83.8%), contempt (75.4%) and sadness (67.9%) in the context of animal cruelty. Considering the bipolarity of these emotions (opposite dyad), it is not surprising that animal welfare activists are fearless (the opposite emotion of anger) in protecting their views and at the same time distrustful (the opposite emotion of trust). The primary dyads of their strongest emotions almost urge, with a strong remorse, to condemn (contempt), which may explain why they assume a significantly lower level of efficiency among investigating authorities and the judiciary.

In our research, those who actively participate in the protection of animals have a less unanimously positive opinion of animal rights NGOs; half of the respondents (50%) consider only some of them to be useful. Presumably, they can also see flaws that are not always obvious to ardent supporters and outsiders. This is significantly different from the image that domestic and international NGOs create of those engaged in the protection of animals [30,31,32,33]. In the literature, the assessment of the animal welfare situation primarily refers to the treatment of animals, professional challenges and tasks [34,35,36,37], and questions related to NGOs are only exceptionally [38] included amongst the problems to be solved [39,40]. Some extreme animal rights activists commit crimes in order to achieve their goals, e.g., against industry and companies that use animal testing [41,42,43]. It may be considered indirect social feedback that, in the United Kingdom, (which has a strong reputation for the protection of animals), the moderate animal rights movements have more members with a budget larger by an order of magnitude than the radical ones [44].

Official bodies do not enjoy support from society everywhere. Finnish official veterinarians are often threatened [45], the members of American animal police forces feel like “second-rate” police officers and are dissatisfied with the work of the courts [46]. The cooperation between NGOs and authorities also serves to protect animals and the community [47,48]. After the experience of using a two-month protocol related to animal protection training at the Environmental Military Police of the State of São Paulo, 200 officers were able to evaluate incidents more effectively and recorded better quality data about the cases [49]. The effect of protocols on animal protection procedures is clearly beneficial [50,51]; so is the creation of large databases [52], so criminal statistics from the United States can be queried [53]. In 2020, 1.5% of crimes (11,566 cases) qualified as animal cruelty in the US.

Emotions also play a role on the law enforcement side, although there are other influences, professional and otherwise [54,55]. We compared the two groups’ perspectives on the relative importance of animal cruelty within criminal offences.

Our findings show that the majority (69.7%) of police authorities consider animal cruelty to be a more serious crime than the average. This rate is 98.7% for animal welfare activists. Almost five times as many activists (76.0%) consider it particularly important than police officers (16.2%). As the investigators gain experience in cases initiated upon the suspicion of animal cruelty, an increasing proportion of them (16.0% after up to five cases, 26.8% after six to twenty cases, and 42.8% after more than twenty cases) consider it an average crime. This can even be interpreted as the opposite of the behaviour expected on the basis of the outcomes of the international literature. It is to be noted that, with more experience, police officers consider animal cruelty to be an average crime; however, it is not clear what this increase stems from. We are not aware of how a police officer prioritised crimes prior to gaining experience, and “average” may mean something different to each individual. Internationally, animal cruelty is a particularly important crime, primarily due to its actual connection with violence against people, which is a view shared by the investigating authorities [56,57], human [58] and animal [59] protection and professional organizations [60]. Public opinion is informed from newspaper articles [61] and books [62], and social media are also used, even by Scotland Yard [63]. Those with a criminal record are significantly more likely (34.5%) to be indifferent to suspicion than those with a clean criminal record (9.5%). The latter object to this more often (18.9%) than those with a criminal record (5.3%).

Among the crimes associated with animal cruelty, illegal animal fighting, theft and illegal gambling are encountered frequently by some investigating authorities. In the literature, another survey mentions robbery, harassment, sexual violence, deliberate arson, robbery, arson and threatening behaviour [64]. The most common psychiatric disorders among people who involved in animal abuse were chronic alcohol consumption disorders (63.7%), domestic violence (53.9%), lifelong nicotine addiction (36.2%) and antisocial personality disorders (35.8%) [65]. A survey of two hundred prosecutors in the USA examined which factors help or hinder the prosecution of such cases. Cruelty (78.7%), animal fighting (30.9%) and zoophilic acts (21.2%) were the most common forms of perpetration. Associated crimes were intimate partner violence (23.5%), drug abuse (22.9%), illegal possession of weapons (13.5%), child or elderly abuse (10.6%), gambling (10%), physical assault (9.4%) and gang-related activities (5.9%), as well as arson, burglary and theft (2.9%).

The public expects animal abusers to be held accountable. The investigators need special training because successful prosecution depends on expertise, and the importance of veterinary forensic expertise is increasing [66]. Our findings show that a third of the accused pleaded guilty and 4.1% were acquitted. This is very similar to the results of a previous Hungarian survey, in which a 3% acquittal rate was found out of 591 finally closed procedures in 185 Hungarian prosecutors’ offices [67]. According to our present survey, the main obstacle to increasing effectiveness is the limited investigation capacity. The main source of help could be cooperation with other authorities; there is no lack of technology in practice. Police investigators named the same necessary conditions almost unanimously, regardless of the professional experience they had of investigating animal cruelty.

In our study, a fraction of animal welfare activists (3.3%) supposed that proceedings were initiated upon about three-quarters of reports; the vast majority (81.3%) believed that it occurred in only a quarter of the cases.

The highest proportion of responses to questionnaires came from Budapest and its surroundings (Central Hungary), where—according to public opinion—the most assertive animal welfare activists live. This is supported by the fact that, according to our survey, anonymous reports are the rarest in this area. Yet, the least of them believed it likely (15.3%) that the authorities would take action. In Transdanubia, animal welfare activists were almost three times more likely (45.5%) to believe that the police would act on a report in half of the cases. In reality, there was no significant difference between the two areas (or even countrywide) in the proportion of cases reported to the authorities, but it was far higher than the animal welfare activists assumed.

Police offices actually initiated an investigation in 72.0% of reports, and a decision was usually (75.6%) made within a week. Only less than a tenth (7.0%) of the cases took longer than a month for a decision to be made. The more experienced the investigator is in this field, the sooner decision-making takes place and the more the proportion of protracted decisions decreases. Procedures were usually terminated (75.8%) within 3 months or reached the prosecution phase within a year at roughly the same rate (78.1%). Furthermore, 2.0% of animal welfare activists assumed that 100% of the accused would be convicted, and 90.0% believed that only a quarter of them would be convicted. A previous study showed that in Hungary, the courts convicted 95.0% of the cases and acquitted 1.0% of the accused for lack of conclusive evidence [68].

Our results show that, according to the animal welfare activists, the punishments have either no (76.0%) or little (18.0%) deterrent effect, and in their opinion, the main reason for this (76.5%) is that the convicts do not receive a prison sentence to be served immediately. Fines, education and the obligation to perform reparative work with or for animals, or even extreme forms of punishment (from humiliating physical abuse to public execution) would be necessary, according to some animal welfare activists. It is important to note that a non-suspended prison sentence can easily become counterproductive without appropriate reintegration and rehabilitation efforts, due to the criminalisation experienced day by day within the walls of the penal institutions, and due to so-called prison harms [69]; as such, it is recommended to impose it only after extensive consideration, if the objective of punishment cannot be achieved in any other way, taking the specifics of the particular case into account. This dissatisfaction is not an exclusively Hungarian feature. For decades, animal rights activists have seen a gap between the wording and the enforcement of animal protection laws internationally, causing people to criticise authorities and governments [70]; penalties are not considered sufficient [71]. In Australia (New South Wales), between January 1996 and December 2000, only 3% of animal abusers were imprisoned. In addition, 80.0% served four months or less; 20.0% of the accused were acquitted and 75.0% of violators were sanctioned (98.0% were sentenced to a fine, 2.0% to community service). There was hardly any noticeable difference in the severity of the sentences imposed [72]. The public expects greater engagement by the courts to advance animal welfare in Australia [73]. In Poland, perpetrators are seldom prosecuted and, even when they are, they are rarely severely punished. According to the same study, Polish police are sometimes slow and unprofessional, and animal welfare organizations play an important role in detecting cases of animal cruelty [74].

Regarding punishments, it is easier to criticise than to develop an effective practical method. An imaginary case was presented to university students, for which they made strongly emotional punishment proposals, in which it was the age of the perpetrator that had a decisive effect rather than the act itself or the animal’s actual suffering [75]. In other experiments, gender and species of animal were the two strongest predictors, which suggests that participants tend to focus on the object of the crime, i.e., the animal, rather than the crime, and then use their own beliefs about that animal to punish. Instead of recommending rehabilitative measures, they wanted to restrict the offender’s ability to keep animals in the future, as well as impose fines and imprisonment. Women with agricultural experience judged the case more severely, men more leniently [76]. In both human and animal abuse, the participant’s mood and the victim’s similarity to the participant influence the participant’s desire to help the victim and punish the perpetrator. In the event of the respondent being in a better mood and considering abuse of animals closer to humans, a more severe punishment was recommended [77].

In Australia, the maximum sentence for animal welfare offences was amended and doubled in 2008. This demonstrates the legislator’s intent regarding penalties, but studies suggest that the courts do not reflect the legislative intent behind increased penalties. On the other hand, since these amendments, the average punishment has doubled [78]. Raising questions of social justice, an alternative solution could be a transition from punishment-oriented approaches to support-based models of animal regulation [79]. The evolution of disruptive and criminal behaviour takes place in a progressive manner and detecting animal cruelty would make earlier and more effective intervention possible [80].

## 5. Conclusions

Most of the investigating authorities consider it important or particularly important to investigate suspicions of animal cruelty; in the vast majority of cases, they decide to initiate proceedings within a week, which can usually lead to indictment within a year. Proceedings are initiated based on more than three-quarters of the reports, but the public is not informed of this, and even the animal welfare NGOs, which are most interested in it, underestimated this proportion by several orders of magnitude. The investigation and the indictment are extremely thorough; the court convicts almost all the defendants, but both animal welfare activists’ and the public have an almost opposite idea about this. The international literature has proved the connection between animal cruelty and violence against people many times. This constitutes sufficient reason for the police to treat animal cruelty as a crime taking priority on professional grounds. However, the strong and animal-centred sentiments of animal welfare activists are accompanied by a tendency to be judgmental and distrustful, which highlights the need for professional and detailed information to be provided to the public.

The police basically gave answers based on data, while the animal welfare activists, for objective reasons, did not have data on the proceedings and therefore expressed their assumptions, opinions and feelings. A comparison of the answers to the same or similar questions mainly indicates that there is a lack of effective dissemination of information to the public in this area.

## Figures and Tables

**Table 1:** Crimes associated with the felony of animal cruelty (*n* = 99).

Associated Crimes	Often	Rarely	Exceptionally
Prohibited animal fights	7	14	8
Theft	3	11	13
Illegal gambling	3	3	1
Firearms offences	2	10	5
Drug offences	2	4	3
Assault and battery	1	6	11
Robbery	1	1	1
Usury	0	2	3
Prostitution	0	1	1
Other crimes	3	2	5

## Data Availability

The data presented in this study are available on request from the corresponding author without undue reservation.

## Supplementary Materials

The following supporting information can be downloaded at: https://www.mdpi.com/article/10.3390/ani13071224/s1, Questionnaire S1: Police; Questionnaire S2: NGO.
