# Supplementary material for: Comparison of Police Data on Animal Cruelty and the Perception of Animal Welfare NGOs in Hungary
Source: Animals (Basel). 2023 Mar 31;13(7):1224. doi: 10.3390/ani13071224 (PMC10093255; doi:10.3390/ani13071224)
Supplement: Supplementary file 1 [file animals-13-01224-s001.zip › Questionnaire S2.pdf]

Animal rights activists' opinions on animal cruelty

Dear madam, dear sir,
As part of our PhD work on proving the crime of cruelty
to animals committed by ill-treatment, we conducted a survey of prosecutors and judges to investigate the path to convictions. A publication presenting the results has been released. We now want to find answers to the question of how those most committed in society perceive all forms of animal cruelty. The questionnaires are anonymous and no personal data are collected. Please help this research by giving us your views. The results will be published in a freely accessible format..
Yours faithfully
Gábor Lorászkó, forensic expert

1. 1. What proportion of reports of animal cruelty do you think result in a procedure being initiated?

Soronként csak egy oválist jelöljön be.

- In about a quarter of cases
- In about half of cases
- In about three quarters of cases
- In every case.
- Egyéb: ______________________

2. 2. What proportion of people who have been charged with animal cruelty do you think are convicted in court?

Soronként csak egy oválist jelöljön be.

- In about a quarter of cases
- In about half of cases
- In about three quarters of cases
- In almost every case
- Egyéb: ______________________

3. 3. In your opinion, do punishments have the necessary deterrent effect on perpetrators of the criminal offence of animal cruelty?

Soronként csak egy oválist jelöljön be.

- Yes, they definitely have deterrent effect.
- Their deterrent effect is slight.
- They have practically no deterrent effect.

4. 4. In your opinion, what would the MOST EFFECTIVE way of improving the deterrent effect of punishments against those who commit crimes of animal cruelty?

Soronként csak egy oválist jelöljön be.

- By increasing the maximum term of imprisonment.
- By serving a custodial sentence rather than a suspended sentence.
- By public naming and shaming.
- By another method instead: please explain at the next question.

5. 5. In what ways other than those listed above could the deterrent effect be strengthened against perpetrators of the crime of animal cruelty?

6. 6. How important do you think it is that, during the investigation of a crime of animal cruelty, the special physiological needs of the given animal species should also be taken into account?

Soronként csak egy oválist jelöljön be.

- I don't think it's necessary; it's enough if we humans put ourselves in the place of the given animal.
- I think it's sometimes necessary, especially in cases of less well-known animal species but, basically, what isn't good for my needs is, in my opinion, just as bad for animals
- I think it's needed, because without an exact knowledge of the needs of a specific species, it isn't possible to determine whether or not the examined outcome (abuse or living conditions) caused the animal to suffer.

7. 7. What is your opinion of news in the media about animal cruelty?

Soronként csak egy oválist jelöljön be.

- I generally think it's first-hand, reliable information. It's suitable for getting general information and for attitude shaping.
- I often come across indirect and distorted content that wants to influence my opinion.
- I consider it as as mixed tabloid news; it's interesting rather than a source that's suitable for obtaining information.
- Depending on the case and the quality of the information, the news accordingly influences me or leaves me cold.

8. 8. How do you treat messages or content published on social media?

Soronként csak egy oválist jelöljön be.

- I comment regularly, and I also share the news .
- I regularly express my displeasure and indignation; I sometimes comment on the news, and I occasionally share it.
- I express my displeasure and indignation, but I don't comment and I don't share.
- I generally I don't form a definite opinion as a result of individual pieces of news because I don't consider myself sufficiently well-informed.
- I'm not interested in news related to animal cruelty.

9. 9. In which country did you acquire the experience related to your answers and where do you live?

Soronként csak egy oválist jelöljön be.

- Bács-Kiskun
- Baranya
- Békés
- Borsod-Abaúj-Zemplén
- Csongrád-Csanád
- Fejér
- Győr-Moson-Sopron
- Hajdú-Bihar
- Heves
- Jász-Nagykun-Szolnok
- Komárom-Esztergom
- Nógrád
- Pest
- Somogy
- Szabolcs-Szatmár-Bereg
- Tolna
- Vas
- Veszprém
- Zala
- Budapest

10. 10. Do you have any relationship with an animal rights NGO?

Soronként csak egy oválist jelöljön be.

- Yes, I often take part in animal rights work or help them with donations.
- Yes, and I support their work with my opinion.
- No.

11. 11. What do you think about animal rights NGOs?

Soronként csak egy oválist jelöljön be.

- Their activities are of outstanding value.
- The majority of them are expressly useful, and their self-sacrificing work really is protecting animals.
- Some of them perform valuable work, some of them don't.
- I don't consider them to be important actors in animal protection.

12. 12. What is your biological gender?

Soronként csak egy oválist jelöljön be.

- Female
- Male

13. 13. What is your age group?

Soronként csak egy oválist jelöljön be.

- <18
- 18-30
- 31-50
- 51-70
- 71<

14. 14. To what extent do you consider animal cruelty to be an important crime?

Soronként csak egy oválist jelöljön be.

- I classify it as less important.
- It's not a crime with an outstanding role; it's one of the many to be prosecuted.
- I consider it as socially more important.
- I consider it a crime of outstanding importance.

15. 15. What emotions are triggered in you when someone causes suffering to an animal?

Soronként csak egy oválist jelöljön be.

	none	a little	middling	a lot
**interest, expectation, vigilance**	⬭	⬭	⬭	⬭
**irritation. anger, rage**	⬭	⬭	⬭	⬭
**attention, surprise, astonishment**	⬭	⬭	⬭	⬭
**reflection, sadness, sorrow**	⬭	⬭	⬭	⬭
**boredom, disgust, loathing**	⬭	⬭	⬭	⬭
**unease, fear, dread**	⬭	⬭	⬭	⬭

16. 16. Which form of animal cruelty do you think occurs more often?

Soronként csak egy oválist jelöljön be.

⬭ committed as cruelty
⬭ committed as neglect
⬭ both equally

17. 17. which form of animal cruelty do you think is harder to detect?

Soronként csak egy oválist jelöljön be.

- animal cruelty
- animal neglect
- both equally

18. 18. What do you think is the standard of animal protection in Hungary nowadays?

Soronként csak egy oválist jelöljön be.

- low
- moderate
- high

19. 19. In your opinion, which actor should MOST improve in order for the standard of animal protection to improve?

Soronként csak egy oválist jelöljön be.

- Animal protection NGOs.
- Society's culture of looking after animals.
- Animal protection education in schools.
- The investigating authorities.
- The authorities involved in criminal procedures.
- Education on being a responsible animal owner within the family.

Ezt a tartalmat nem a Google hozta létre, és nem is hagyta azt jóvá.

Google Űrlapok
