# Supplementary material for: Comparison of Police Data on Animal Cruelty and the Perception of Animal Welfare NGOs in Hungary
Source: Animals (Basel). 2023 Mar 31;13(7):1224. doi: 10.3390/ani13071224 (PMC10093255; doi:10.3390/ani13071224)
Supplement: Supplementary file 1 [file animals-13-01224-s001.zip › Questionnaire S1.pdf]

Police

Dear police officers

It seems to be a common perception among the
public that those who commit animal cruelty often go unpunished. However, our research in prosecutors' offices and courts has shown the opposite to be true:
the probability of a conviction is 97% and of acquittal due to the absence of a criminal offence is only 1%. The cases that are brought to court are therefore very carefully prepared. The
questions we are asking now are to find out what happens to the cases that do not reach prosecution. Only you know that. Learning about your experiences may not only help us understand the actual situation but also can improve the
efficiency of procedures; and we ask for your cooperation for a few minutes.
Yours faithfully: Dr. Gábor Lorászkó, forensic expert

*Kötelező

1. 1. In which county did you acquire the experience to which your answers relate?

Soronként csak egy oválist jelöljön be.

- Bács-Kiskun
- Baranya
- Békés
- Borsod-Abaúj-Zemplén
- Csongrád-Csanád
- Fejér
- Győr-Moson-Sopron
- Hajdú-Bihar
- Heves
- Jász-Nagykun-Szolnok
- Komárom-Esztergom
- Nógrád
- Pest
- Somogy
- Szabolcs-Szatmár-Bereg
- Tolna
- Vas
- Veszprém
- Zala
- Budapest

2. 2. From how many procedures did you acquire the experience to which your answers relate?

3. 3. How are procedures initiated?

Soronként csak egy oválist jelöljön be.

	most often	not often	rarely	exceptionally	never
**Anonymous report**	◯	◯	◯	◯	◯
**Reported by a citizen identifying themself**	◯	◯	◯	◯	◯
**Reported by an NGO**	◯	◯	◯	◯	◯
**Direct detection by the police**	◯	◯	◯	◯	◯
**Reported by another authority**	◯	◯	◯	◯	◯
**Other**	◯	◯	◯	◯	◯

4. 4. What other ways are there to initiate the procedure? (If you marked "other" in the previous question.)

____

5. 5. To what extent do you consider animal cruelty to be an important crime?

Soronként csak egy oválist jelöljön be.

- ◯ I classify it as less important
- ◯ It doesn't represent an important crime ; it's one of the many to be prosecuted.
- ◯ I consider it as one of the more socially important ones.
- ◯ I consider it an extremely important crime.

6. 6. What is the reason for not prosecuting?

Soronként csak egy oválist jelöljön be.

- the report is frivolous
- the act or case does not raise suspicion of a criminal offence
- there is too little data to initiate a procedure
- statute of limitation
- Egyéb: ______________________

7. 7. Do you avail of a forensic expert if you want to decide whether to initiate a procedure at all?

Soronként csak egy oválist jelöljön be.

- never
- exceptionally
- not usually
- yes, usually
- always

8. 8. How long does it usually take for the decision to close the case and not open an investigation?

Soronként csak egy oválist jelöljön be.

- a week or less
- 1-4 weeks
- 1-2 months
- more

9. 9. What percentage of reports lead to a procedure?

____

10. 10. Which form of animal cruelty do you think occurs more often?

Soronként csak egy oválist jelöljön be.

- committed as cruelty
- committed as neglect
- both equally

11. 11. Which form of animal cruelty do you think is harder to detect?

Soronként csak egy oválist jelöljön be.

- animal cruelty
- animal neglect
- both equally

12. 12. What additional criminal offences emerge during a procedure initiated on suspicion of animal abuse? *

Válassza ki az összeset, amely érvényes.

	most often	not often	rarely	exceptionally	never
**drug trafficking**	☐	☐	☐	☐	☐
**theft**	☐	☐	☐	☐	☐
**misuse of firearms or ammunition**	☐	☐	☐	☐	☐
**Facilitating prostitution**	☐	☐	☐	☐	☐
**robbery**	☐	☐	☐	☐	☐
**causing bodily harm**	☐	☐	☐	☐	☐
**organising games of chance**	☐	☐	☐	☐	☐
**crimes of usury**	☐	☐	☐	☐	☐
**others: l. next question**	☐	☐	☐	☐	☐

13. 13. What additional criminal offences arise during a procedure initiated on suspicion of animal cruelty other than those listed above?

____

14. 14. Approximately what percentage of procedures are terminated without prosecution?

____

15. 15. For which modus operandi (in your opinion) are terminations (without prosecution) more frequent?

Soronként csak egy oválist jelöljön be.

- committed as cruelty
- committed as neglect
- both committed equally often

16. 16. Do you avail of a forensic expert if you ever want to decide whether to terminate a procedure?

Soronként csak egy oválist jelöljön be.

- never
- exceptionally
- not usually
- yes, usually
- always

17. 17. How much time typically elapses before a decision to terminate the procedure is made?

Soronként csak egy oválist jelöljön be.

- a month or less
- a month or two
- 3-4 months
- 5-6 months
- 6-12 months
- more

18. 18. How circumstantial is the investigation of the individual factual elements?

Soronként csak egy oválist jelöljön be.

	self-evident	easy	somewhat circumstantial	difficult
**Whether the activity can be classified as neglect**				
**Whether the activity can be classified as abuse**				
**Whether the activity was justified**				
**Whether the activity would cause long-term damage to health**				
**Whether the death of the animal was possible**				
**Whether extreme suffering occurred**				

19. 19. How long does it usually take for a case to reach indictment?

Soronként csak egy oválist jelöljön be.

- 1-3 months
- 3-6 months
- 6-12 months
- 1-1.5 years
- more

20. 20. What kind of reaction does the suspicion of animal cruelty usually elicit from the suspected perpetrator, who has NO criminal record?

Soronként csak egy oválist jelöljön be.

- They usually take the suspicion with complete indifference.
- They find it a bit uncomfortable, but it doesn't bother them.
- As usual with non-serious crimes.
- They protest. They find suspicion unpleasant and embarrassing.
- They react with violent protest or show intense shame.
- The reactions are extremely different, it is not possible to indicate what they usually are.

21. 21. What kind of reaction does the suspicion of animal cruelty usually elicit from the suspected perpetrator, who has CRIMINAL record?

Soronként csak egy oválist jelöljön be.

- They usually take the suspicion with complete indifference.
- They find it a bit uncomfortable, but it doesn't bother them.
- As usual with non-serious crimes.
- They protest. They find suspicion unpleasant and embarrassing.
- They react with violent protest or show intense shame.
- The reactions are extremely different, it is not possible to indicate what they usually are.

22. 22. What would be needed to increase the efficiency of procedures?

Válassza ki az összeset, amely érvényes.

- [ ] More detailed and accurate reports
- [ ] Greater investigative capacity (personnel, resources, available time).
- [ ] Greater social cooperation during the investigation and the procedure.
- [ ] Professional cooperation with other authorities (veterinary and animal welfare authorities, notaries, human health).
- [ ] More forensic experts
- [ ] Closer cooperation with animal protection NGOs.
- [ ] Higher level technical equipment.
- [ ] Specialised training on animal cruelty..
- [ ] Other: I next question

23. 23. Please write here if you have anything to add to the previous question that would improve the effectiveness of the investigating authority:

____

Ezt a tartalmat nem a Google hozta létre, és nem is hagyta azt jóvá.

Google Űrlapok
